# RNA-Seq Transcriptome Profiling of the Queen Scallop (*Aequipecten opercularis*) Digestive Gland after Exposure to Domoic Acid-Producing *Pseudo-nitzschia*

**DOI:** 10.3390/toxins11020097

**Published:** 2019-02-06

**Authors:** Pablo Ventoso, Antonio J. Pazos, M. Luz Pérez-Parallé, Juan Blanco, Juan C. Triviño, José L. Sánchez

**Affiliations:** 1 Departamento de Bioquímica y Biología Molecular, Instituto de Acuicultura, Universidade de Santiago de Compostela, 15782 Santiago de Compostela, Spain; pabloventoso24@hotmail.com (P.V.); luz.perez-paralle@usc.es (M.L.P.-P.); joseluis.sanchez@usc.es (J.L.S.); 2 Centro de Investigacións Mariñas, Xunta de Galicia, Pedras de Corón s/n Apdo 13, 36620 Vilanova de Arousa, Spain; juan.carlos.blanco.perez@xunta.gal; 3 Sistemas Genómicos, Ronda G. Marconi 6, Paterna, 46980 Valencia, Spain; jc.trivino@sistemasgenomicos.com

**Keywords:** amnesic shellfish poisoning (ASP), domoic acid, bivalves, *Aequipecten opercularis*, scallop, RNA-seq, transcriptome, differential expression, qPCR, oxidative stress

## Abstract

Some species of the genus *Pseudo-nitzschia* produce the toxin domoic acid, which causes amnesic shellfish poisoning (ASP). Given that bivalve mollusks are filter feeders, they can accumulate these toxins in their tissues. To elucidate the transcriptional response of the queen scallop *Aequipecten opercularis* after exposure to domoic acid-producing *Pseudo-nitzschia*, the digestive gland transcriptome was de novo assembled using an Illumina HiSeq 2000 platform. Then, a differential gene expression analysis was performed. After the assembly, 142,137 unigenes were obtained, and a total of 10,144 genes were differentially expressed in the groups exposed to the toxin. Functional enrichment analysis found that 374 Pfam (protein families database) domains were significantly enriched. The C1q domain, the C-type lectin, the major facilitator superfamily, the immunoglobulin domain, and the cytochrome P450 were among the most enriched Pfam domains. Protein network analysis showed a small number of highly connected nodes involved in specific functions: proteasome components, mitochondrial ribosomal proteins, protein translocases of mitochondrial membranes, cytochromes P450, and glutathione S-transferases. The results suggest that exposure to domoic acid-producing organisms causes oxidative stress and mitochondrial dysfunction. The transcriptional response counteracts these effects with the up-regulation of genes coding for some mitochondrial proteins, proteasome components, and antioxidant enzymes (glutathione S-transferases, thioredoxins, glutaredoxins, and copper/zinc superoxide dismutases).

## 1. Introduction

The amnesic shellfish poisoning (ASP) toxin, domoic acid, is produced by some species of the genera *Pseudo-nitzschia* and *Nitzschia* [1,2]. The prevalence of domoic acid and toxic diatoms seems to have increased worldwide [1]. Domoic acid is a tricarboxylic amino acid that resembles glutamic acid and is a potent glutamate receptor agonist [3,4]. Bivalve mollusks are filter feeders, and during harmful algal blooms, they can accumulate toxins in their tissues. This is why they are the primary vectors for causing ASP in humans [1,5,6].

Domoic acid depuration time in bivalves is species-specific and can differ largely from one species to another [7]. Mussels of the genus *Mytilus* [8,9,10,11] and the oyster *Crassostrea gigas* [9] rapidly eliminate domoic acid, while the king scallop *Pecten maximus* [7] and the razor clam *Siliqua patula* [12] are very slow domoic acid depurators. In mussels and scallops, the digestive gland is the tissue with the highest domoic acid concentration [7,10,13,14]. Mauriz and Blanco [15] suggested that the very low depuration rate of *P. maximus* could be due to the lack of an efficient transmembrane transporter. Unlike the king scallop, in the queen scallop (*Aequipecten opercularis*) the depuration rate is fast [15].

Domoic acid is excitotoxic in the central nervous system of mammals and other vertebrates [3,4], but its putative effects on invertebrates have been less studied. Although it seems that domoic acid-producing organisms are not toxic to shellfish (or at least not highly toxic), they can exert several physiological and sublethal effects on marine bivalves [16,17,18,19,20]. Some of these effects include DNA damage in mussels [16], stress response characterized by shell closure, hemolymph acidosis, hypoxia, an increase in the number and activity of hemocytes in the oyster *C. gigas* [17,18], reduced larval growth in *P. maximus* [19], and negative impacts on growth rate and survival in juvenile king scallops [20]. Some authors have suggested that although several harmful algae toxins do not affect the survival of bivalve mollusks, they provoke oxidative stress [21,22,23]. However, the molecular mechanisms that cause oxidative stress are poorly understood, and furthermore, domoic acid causes oxidative stress in the vertebrate central nervous system [24,25,26,27]. The works cited above [16,17,18,19,20,21] analyzed several physiological and biochemical parameters after exposure to domoic acid but did not study the gene expression patterns in both exposed and non-exposed bivalves.

There are some publications regarding the gene expression changes associated with exposure to domoic acid in vertebrates [24,28,29,30]. The transcriptome response was dependent on the dose and the exposure duration; among the differentially expressed genes were those involved in transcription (transcription factors), signal transduction, ion transport, generalized stress response, mitochondrial function, inflammatory response, DNA damage, apoptosis, neurological function, and neuroprotection [24,28,29,30]. In a previous work, we studied (by RNA-seq) the effects of domoic acid-containing *Pseudo-nitzschia* on gene expression in the mussel *Mytilus galloprovincialis* [31], and to our knowledge, this is the only published work about the transcriptional effects of domoic acid exposure on mollusks. As stated before, among the bivalves there are large interspecific differences in the domoic acid depuration rate [7,8,9,10,11,12]. It is therefore necessary to carry out gene expression studies on different species.

Understanding the molecular mechanisms of domoic acid uptake and elimination in bivalves and how the toxin (and the toxin-producing species) affects gene expression are two knowledge gaps in this field. The aim of the present work is to contribute to filling these gaps by means of a transcriptomic approach. First, the whole transcriptome of the *A. opercularis* digestive gland was de novo assembled, and then, we analyzed by RNA-seq the transcriptional changes after exposure to domoic acid-producing *Pseudo-nitzschia*. This approach can provide some clues regarding the biological and molecular processes altered by domoic acid. The transcriptomic approach has been successfully employed to uncover the genetic response of bivalves to diarrhetic shellfish poisoning (DSP) and paralytic shellfish poisoning (PSP) toxins and also to identify the genes putatively involved in detoxification processes [32,33,34,35,36].

## 2. Results

### 2.1. Domoic Acid Content in the Digestive Gland of A. opercularis

A group of six scallops sampled on April 9 from the tank (group DB) had an average domoic acid content of 1361 ± 804 ng/g digestive gland wet weight, while a group of six scallops sampled on April 17 from the raft (group DA) had an average domoic acid content of 6680 ± 1661 ng/g digestive gland wet weight (Table 1). In the six control scallops sampled on May 12 from the tank (group C), the domoic acid levels were below the limit of quantification (BLOQ, Table 1). The total scallop wet weights and digestive gland (DG) wet weights are shown in Table 1 and Table S1.

### 2.2. Sequencing and de novo Assembly

After the de novo assembly, the transcripts were clustered (homology >90%) to reduce redundancy. Thus, 142,137 unigenes were obtained (Table 2). The minimum, maximum, and mean contig lengths were 200, 17,867, and 1343.9 bp, while the N50 contig length was 1845 bp (Table 2). The raw data are accessible from the NCBI Sequence Read Archive (Project PRJNA508885, sample accession numbers from SAMN10537388 to SAMN10537405).

### 2.3. Differential Expression, Functional Annotation, and Functional Enrichment Analysis

**Figure 1:**
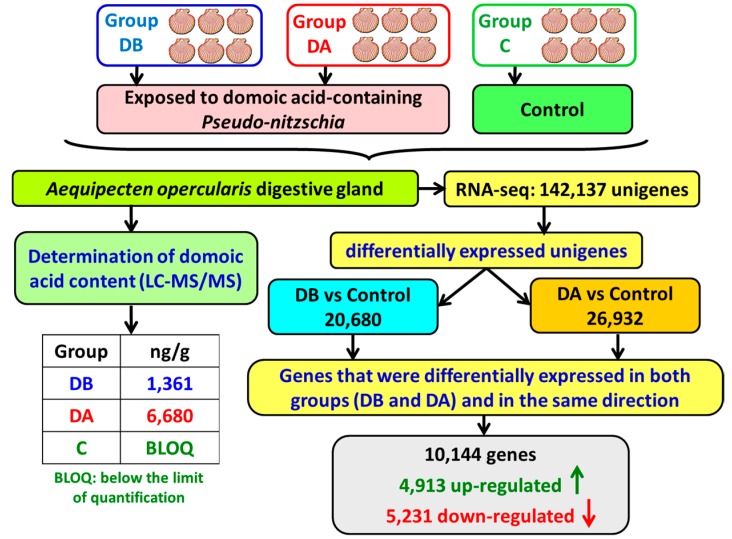
Scheme of the differential expression results obtained in *A. opercularis* digestive glands after exposure to domoic acid-producing *Pseudo-nitzschia*.

A total of 26,932 and 20,608 differentially expressed genes (DEGs) were detected in group DA and in group DB, respectively, when compared to the control (C) group (Figure 1). Genes that were differentially expressed in both groups (the groups that had accumulated domoic acid) and in the same direction (either down- or up-regulation) were selected for further study: 10,144 genes, including 4913 up-regulated and 5231 down-regulated (Figure 1; Table 3 and Table 4; Files S1 and S2). The top 25 significantly up-regulated genes are listed in Table 3. Genes coding for fatty acid-binding proteins and cytosolic sulfotransferases were among the top up-regulated genes (Table 3). Most of the top 25 down-regulated genes do not have a Blastx hit (Table 4; File S2).

A summary of the functional annotation results is shown in Table 5. After functional enrichment performed using the Pfam annotations, 374 domains were found to be significantly (false discovery rate (FDR)-adjusted *p*-value <0.05) enriched in the DEGs (Table 6; File S3). The C-type lectin, the RNA recognition motifs, the major facilitator superfamily, and the cytochrome P450 were among the most enriched Pfam domains whose genes were mostly up-regulated (Table 6). In addition to the cytochromes P-450, several Pfam domains involved in biotransformation (phase I and phase II metabolism of xenobiotics) were functionally enriched and up-regulated: glutathione S-transferases, sulfotransferases, methyltransferases, and aldehyde dehydrogenases (Table 6; File S3). By contrast, most of the genes coding for proteins with C1q domains, immunoglobulin domains, tetratricopeptide repeats, and collagen triple helix repeats were down-regulated (Table 6; File S3).

Significantly enriched gene ontology (GO) terms (Fisher’s exact test, FDR-adjusted p-value <0.05) in the biological process (BP), molecular function (MF), and cellular component (CC) categories are displayed in Table 7 (up-regulated DEGs), Table 8 (down-regulated DEGs), and in File S4. A greater number of enriched GO terms was obtained for the up-regulated DEGs (738) than for those down-regulated (229). The analyses identified 426, 198, and 114 enriched GO terms in the BP, MF, and CC categories, respectively, for the up-regulated DEGs (File S4). The top significantly enriched GO terms (classified by FDR) were (Table 7) metabolic process, oxidation-reduction process, and organic substance catabolic process (in the BP category); catalytic activity, oxidoreductase activity and threonine-type peptidase activity (in the MF category); and cytoplasm, proteasome complex, and endopeptidase complex (in the CC category).

On the other hand, the number of enriched GO terms for the down-regulated DEGs (File S4) were 86 (BP), 111 (MF), and 32 (CC). Table 8 shows that the top enriched GO terms were as follows: neurotransmitter transport, regulation of cellular process, and cell communication (in the BP category); neurotransmitter transporter activity, neurotransmitter/sodium symporter activity, and solute/sodium symporter activity (in the MF category); and transcription factor complex, collagen trimer, and cytoskeleton (in the MF category).

**Figure 2:**
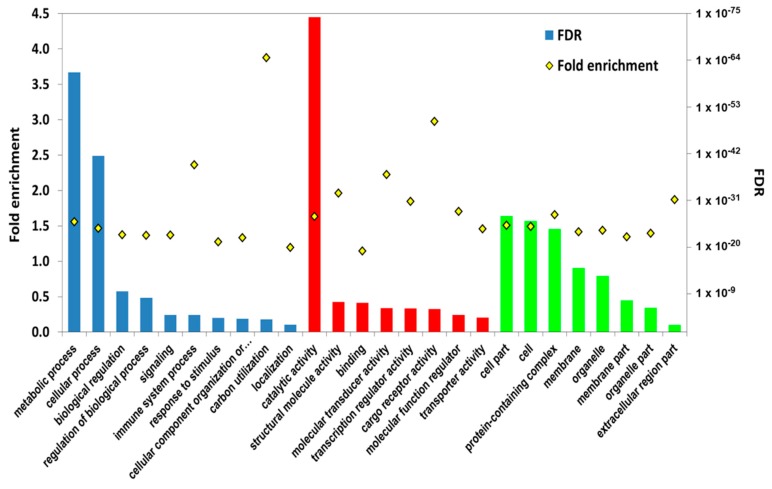
Level-2 enriched gene ontology (GO) terms for the differentially expressed genes in the biological process (BP, blue), molecular function (MF, red), and cellular component (CC, green) categories. FDR: p-value adjusted by FDR. Histograms represent the FDR-adjusted p-value, and the yellow diamonds represent the fold enrichment.

Among the level-2 enriched GO terms (Figure 2; Table S2), the genes in the categories of metabolic process, cellular process, catalytic activity, structural molecule activity, and transporter activity were mainly up-regulated, while most of the genes in the categories of biological regulation, signaling, immune system process, response to stimulus, and transcription regulator activity were down-regulated. File S5 shows the Kyoto Encyclopedia of Genes and Genomes (KEGG) orthologues (KO) of DEGs and of all unigenes.

### 2.4. Protein Network Analysis

**Figure 3:**
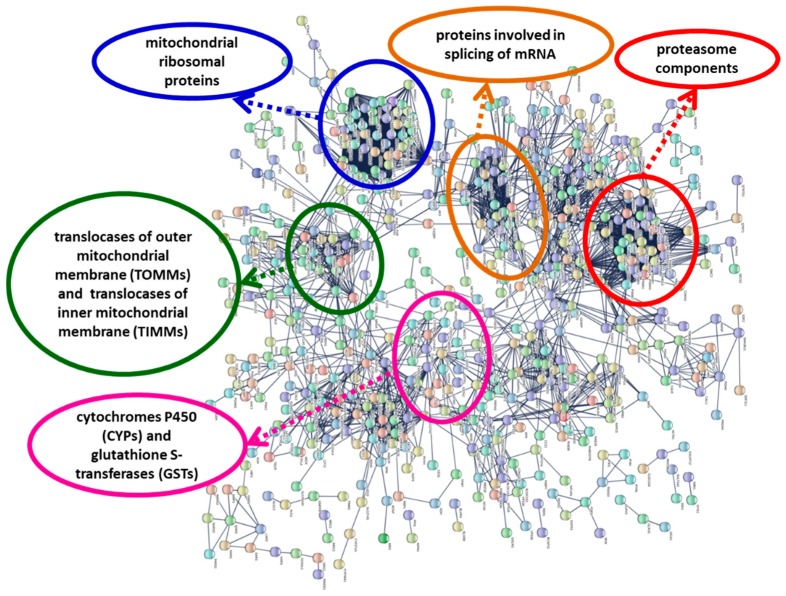
Network showing interactions of proteins coded by genes up-regulated in the present study. Network was constructed using the String 10.5 algorithm and obtained in the highest confidence (0.9) mode. Some highly connected protein nodes are highlighted. Proteins were named according to the human protein name. A full list of protein names is available in Figure S1 and File S6.

Protein–protein interactions can be employed to group and organize all the protein-coding genes in a genome [37]. From the 4913 up-regulated genes, a Blastx search found 931 human homologs in the STRING database. The network obtained in the highest confidence (0.9) mode is enriched in interactions (*p*-value <1 × 10^−16^). The results obtained with the up-regulated DEGs showed a small number of highly connected protein nodes. Each group of proteins is involved in specific biological processes (Figure 3; Figure S1; File S6): degradation of proteins (proteasome components), synthesis of mitochondrial proteins (mitochondrial ribosomal proteins), translocation of cytosolically synthesized mitochondrial preproteins (translocases of outer and inner mitochondrial membrane, TOMMs, and TIMMs), splicing of mRNA (spliceosome components), and phase I and phase II metabolism of xenobiotics (cytochromes P450 and glutathione S-transferases).

From the 5231 down-regulated DEGs, 855 human homologs were found in the STRING database. The network is enriched in interactions (*p*-value <1 × 10^−16^). Components of different types of collagen, heat shock proteins, and proteins involved in cytoskeleton dynamics (Figure S2; File S7) were among the proteins that appeared in the network obtained with the down-regulated DEGs.

### 2.5. Real Time RT-qPCR

**Figure 4:**
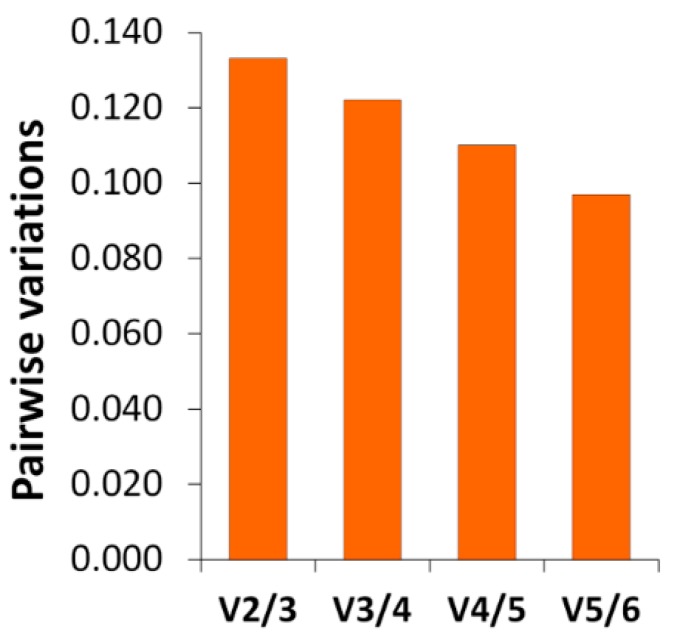
Determination of the optimal number of reference genes for normalization. The pairwise variation (Vn/n+1) was calculated between the normalization factors NFn and NFn+1 (using n or n+1 reference genes respectively) by geNorm software.

The candidate reference genes (*EIF4EBP2*, *RPS4*, *VAMP7*, *RAP1B*, *DNAJ*, and *MYH9*) (Table 9) were selected for their stable expression based on the RNA-seq data. NormFinder stability values ranged from 0.137 to 0.237 and the geNorm average M from 0.339 to 0.584 (Table 10). The standard deviations (SD) of Cq values calculated with BestKeeper were low (0.53–0.69, Table 10). Pairwise variation (Vn/n+1) [38] was used to determine the optimal number of reference genes for normalization. Figure 4 shows that V5/6 attained the minimum pairwise variation value (0.097); therefore, five reference genes were used for normalization [39]: *EIF4EBP2*, *RPS4*, *VAMP7*, *RAP1B*, and *DNAJ*. The least stable reference gene candidate was *MYH9*.

**Figure 5:**
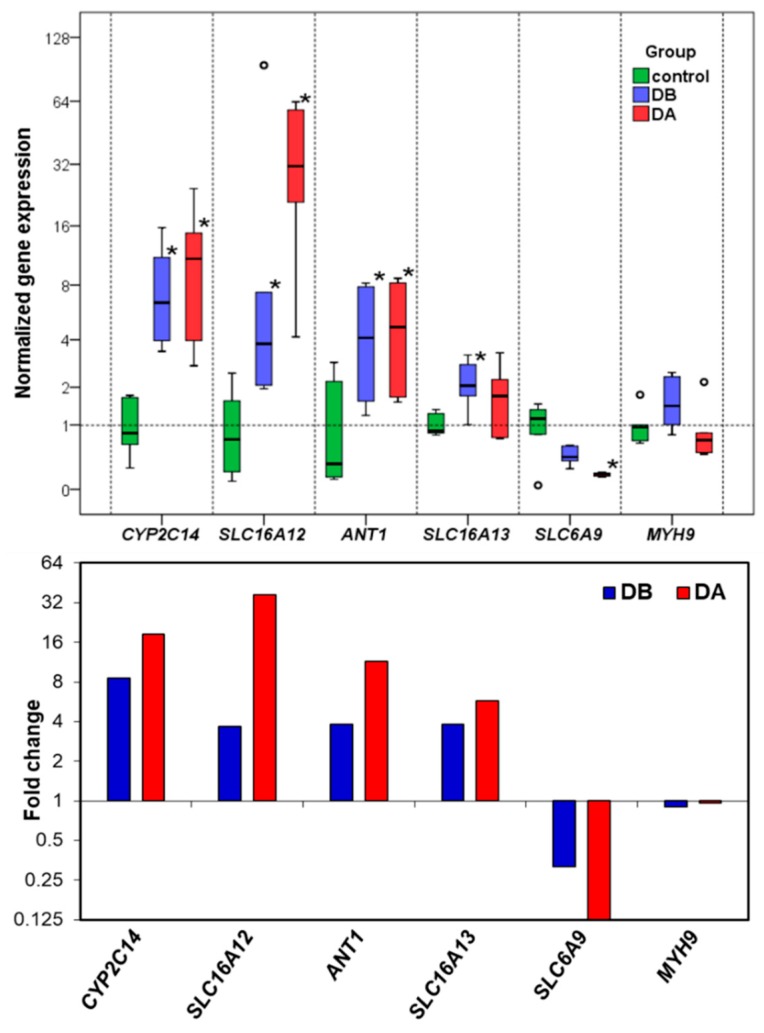
Gene expression. Upper panel: Normalized gene expression in the digestive gland of *A. opercularis* in the presence of domoic acid, as determined by RT-qPCR analyses. The box and whisker plots were obtained using IBM SPSS version 24.0 software. The boxes represent the lower and upper quartiles with medians. The bars represent the ranges for the data (*n* = 6). The circles represent extreme values (more than three box lengths from the end of a box). The statistical analysis was performed using ANOVA and Dunnett’s Two-Tailed *t* Test: *p <0.05. Lower panel: Fold change in relation to the control obtained by RNA-seq.

The normalized gene expression of the five target genes, and the non-selected reference gene (*MYH9*) is displayed in Figure 5. There was good agreement between RT-qPCR (Figure 5, upper panel) and RNA-seq (Figure 5, lower panel). The RNA-seq results showed that *CYP2C14*, *SLC16A12*, *ANT1*, and *SLC16A13* were up-regulated in groups DB and DA in relation to the control; these genes were also up-regulated when the RT-qPCR data were analyzed. The *SLC6A9* gene was down-regulated in groups DB and DA in relation to the control group (Figure 5, lower panel), but the RT-qPCR data showed significant differences only between group DA and the control. The candidate reference gene *MYH9* is not differentially expressed.

## 3. Discussion

There are many studies about the mechanisms of the neurotoxicity of domoic acid in vertebrates (especially mammals), but there is very little knowledge about the putative effects of domoic acid on bivalve mollusks. Several publications showed that domoic acid can exert physiological and sublethal effects on marine bivalves [16,17,18,19,20]. Dizer et al. [16] found that in *Mytilus edulis*, DNA damage was significantly increased after the injection of domoic acid and suggested the existence of genotoxic responses in the cells of digestive glands. It is interesting to point out that DNA repair, cellular response to DNA damage stimulus, and cellular response to stress were among the enriched GO terms in the present work for the up-regulated DEGs (File S4). This could be the transcriptomic response of *A. opercularis* to the putative DNA damage provoked by domoic acid. In *C. gigas*, domoic acid provoked a generalized stress response [17] and an increase in the number and activity of hemocytes [18]. Domoic acid induces oxidative stress in the central nervous system and spinal cord in vertebrates [24,25,26,27]. Furthermore, harmful algae toxins sometimes provoke oxidative stress in bivalves [21,22,23], and Prego-Faraldo et al. [40] found that exposure to the toxic dinoflagellate *Prorocentrum lima* induces the differential expression of genes coding for antioxidant enzymes. The glutathione S-transferase, thioredoxin, glutaredoxin, and copper/zinc superoxide dismutase Pfam domains were functionally enriched in queen scallops (File S3), and these genes were, for the most part, up-regulated; these domains are found in proteins involved in protection against reactive oxygen species (ROS).

In a recent work about the effects of environmental stress on gene transcription in oysters, Anderson et al. [41] proposed a consensus model of sub-cellular stress responses in oysters with the involvement of mitochondria and reactive oxygen species (ROS) production. If the anti-oxidant enzymes and molecular chaperones cannot limit the damage caused by ROS, then the consequences are probably cellular dysfunction and apoptosis [41]. In vertebrates, domoic acid causes mitochondrial dysfunction as a consequence of oxidative stress [4,24,26]. Hiolski et al. [24] suggested the existence of compensatory mitochondrial biogenesis in response to mitochondrial dysfunction. Our results (Figure 3; Figure S1; File S2,S6) showed an up-regulation of genes coding for mitochondrial ribosomal proteins and translocases of the outer and inner mitochondrial membrane (proteins involved in mitochondrial biogenesis); in the protein interaction network, these proteins form highly connected protein nodes (Figure 3; Figure S1)

Up-regulation of proteasome subunits (Figure 3; Figure S1; File S2) is also a possible consequence of oxidative stress [42], because the proteasome is responsible for the selective degradation of oxidized proteins [43]. The 26S proteasome is a protease complex, which is responsible for the regulated degradation of proteins in eukaryotic organisms [42]. The proteasome system can be activated to accomplish the destruction of proteins altered by stress conditions [44]. The proteasome complex and proteasome core complex were two of the most enriched GO terms in the cellular component category for the up-regulated DEGs (Table 7), and the proteasome Pfam domain (PF00227) was also enriched (File S3); the genes coding for proteins with this domain were up-regulated in *Pseudo-nitzschia*-exposed scallops (Files S2 and S3). Proteasome proteins form a group of highly connected nodes in the protein–protein interaction network (Figure 3, Figure S1). In *M. galloprovincialis* exposed to the toxin okadaic acid, there is an up-regulation of several mRNAs involved in proteasome activity [45]. Therefore, the results support the hypothesis that exposure to domoic acid-producing *Pseudo-nitzschia* causes oxidative stress and the impairment of the mitochondrial function in *A. opercularis* and that the transcriptional changes are directed, at least in part, to counteract the stress effects.

The metabolism of xenobiotics (such as toxins) has three phases; phase I (functionalization) and phase II (conjugation) are catalyzed by metabolizing enzymes, while phase III consists of the export from the cell by transmembrane transporter proteins. We found that the Pfam domains of some phase I (cytochromes P450 and aldo-keto reductases) and phase II (glutathione S-transferases and sulfotransferases) drug metabolizing enzymes were functionally enriched, and the genes coding for these enzymes were mostly up-regulated. Cytochromes P450 and glutathione S-transferases constituted a group of highly connected nodes in the protein interaction network (Figure 3, Figure S1). Genes of these families were also up-regulated in mussels (*M. galloprovincialis*) exposed to domoic acid-containing *Pseudo-nitzschia* [31]. Peña-Llopis et al. [46] showed that the treatment of scallops (*P. maximus*) with N-acetylcysteine increased glutathione S-transferase (GST) activity and enabled the scallops to eliminate domoic acid more efficiently. Therefore, it is possible that glutathione S-transferases play a role in domoic acid detoxification. Li et al. [35] found an up-regulation of sulfotransferase genes in the kidney of the Zhikong scallop *Chlamys farreri* after exposure to paralytic shellfish toxin-producing *Alexandrium minutum*. Furthermore, the family of sulfotransferases is significantly expanded in the *C. farreri* genome [35]; the high number of transcripts coding for sulfotransferases in the *A. opercularis* transcriptome is indicative of an expansion of this family in the queen scallop.

The molecular mechanisms of domoic acid absorption and excretion in bivalve mollusks are poorly understood [31]. Mauriz and Blanco [15] found that in the king scallop *P. maximus*, domoic acid is free in the cytosol of the digestive gland and suggested that the low depuration rate of domoic acid in this species could be due to the lack of membrane transporters. Domoic acid is a charged compound and probably needs a transport protein to pass through the plasma membrane, as is the case with glutamic acid [47,48]. This putative transmembrane transporter(s) could therefore play an important role in the absorption and/or the excretion of domoic acid. The results of Kimura et al. [47] suggest that anion exchange transporters are responsible for the transmembrane transport of domoic acid in Caco-2 cell monolayers (which represent the intestinal barrier of mammals); these transporters belong to the solute carrier (*SLC*) superfamily. In *A opercularis*, we found that transmembrane transport and transmembrane transporter activity were two of the enriched GO terms (File S4); furthermore, the major facilitator superfamily (MFS) was one of the most significantly enriched Pfam families (Table 6). Most of the genes belonging to these categories were up-regulated (Table 6; Files S2–S4). MFS is a clan of the SLC superfamily [49], and Hediger et al. [50] reported that the *SLC* gene series included 52 families in the human genome, although it has recently been updated to 65 families [51]. A total of eight *SLC* families (*SLC5*, *SLC16*, *SLC17*, *SLC21*, *SLC22*, *SLC26*, *SLC39*, and *SLC49*) contain up-regulated genes in *A. opercularis* (File S8) and in *M. galloprovincialis* [31] exposed to domoic acid-containing *Pseudo-nitzschia*. The transporter protein(s) putatively involved in the uptake and/or elimination of domoic acid in the digestive gland of bivalve mollusks could be encoded by a gene from one of those families. The families with a higher number of up-regulated genes in both *A. opercularis* and *M. galloprovincialis* were *SLC16* (the monocarboxylate transporters family) and *SLC22* (organic cation/anion/zwitterion transporters). A total of four members of the human *SLC16* gene family encode monocarboxylate transporters, but the substrates of several members are unknown [52]. The *SLC22* family [53] comprises organic cation, zwitterion, and anion transporters (OCTs, OCTNs, and OATs), which participate in the absorption (in the small intestine) and excretion (in the liver and kidney) of xenobiotics and endogenous substances [53]. Unfortunately, the lack of knowledge about the identity of the domoic acid transmembrane transporter(s) in mammals makes it difficult to identify them in bivalve mollusks. Schultz et al. [54] suggested that ATP-binding cassette (ABC) transporters are responsible for the absorption of domoic acid in Dungeness crabs, but in *A. opercularis*, we found only five up-regulated ABC transporters. A similar result was reported by Pazos et al. [31] in *M. galloprovincialis* (with two up-regulated ABC transporters). This contrasts with the high number of up-regulated *SLC* genes found in both bivalves.

Although most of the *SLC* genes differentially expressed in *A. opercularis* were up-regulated, the *SLC6* family (the sodium- and chloride-dependent neurotransmitter transporter family) is an exception, with 48 down-regulated unigenes (File S8). Furthermore, neurotransmitter/sodium symporter activity is one of the most enriched GO terms for the down-regulated genes (Table 8). On the contrary, in *M. galloprovincialis*, two genes from this family were up-regulated and none down-regulated [31]. In *A. opercularis*, the number of genes in this family is very high, and this agrees with results from Li et al. [35], who found that the *SLC6* family is expanded in the *C. farreri* (a scallop) genome in relation to other bivalves.

Harmful algae and biotoxins exert different effects on the immune systems of bivalve mollusks [23,32,55]. Immune response and immune system process were two of the most enriched GO terms for the down-regulated DEGs (File S4), and Pfam families involved in immunological processes were significantly enriched in *A. opercularis* (Table 6; File S3): the C1q domain-containing proteins, the C-type lectin, the fibrinogen beta and gamma chains C-terminal globular domain, the immunoglobulin domain, and tumor necrosis factors (TNF). Except for the C-type lectins, the genes in these categories were mainly down-regulated (Table 6; Files S2 and S3). Differentially expressed genes from these families have been found in several bivalve mollusks after exposure to different biotoxins [31,32,33,34,36,56,57]. Hégaret et al. [23] found that some harmful algae provoked a stimulation of immune function of bivalve hemocytes, while others were immunosuppressive. The C1q domain containing proteins and the C-type lectins are particularly abundant in the digestive glands of bivalves [58,59]. The C1q domain-containing proteins are indispensable in the innate immune systems of invertebrates [60] and could be involved in several functions, such as activation of the complement pathway, cell adhesion, pathogen recognition, response to pollutants, and apoptosis [58,60,61]. An expansion of the genes coding for proteins containing the C1q domain was found in several bivalves [58,61,62,63]. For example, 321 C1q domain-containing proteins are encoded by the *C gigas* genome [62]; this represents approximately 10-fold more than the Ciq proteins encoded by the *Homo sapiens* genome [62]. Some genes coding for C1q domain-containing proteins were down-regulated in *M. galloprovincialis* fed with toxigenic strains of *Alexandrium minutum* [34]. The C-type lectins are characterized by a calcium-dependent carbohydrate recognition domain and participate in pathogen recognition and in innate immunity in bivalves [59], but they can also perform non-immune functions; for example, a role in efficient food particle sorting (food recognition) was found in the oyster *Crassostrea virginica* [64]. There is a high number of genes coding for C-type lectins in bivalve mollusks [59,65]. Most of the genes coding for C-type lectins were up-regulated in *A opercularis* (Table 6; Files S2 and S3) and *M. galloprovincialis* [31] after exposure to domoic acid-producing *Pseudo-nitzschia*; this agrees with the up-regulation of C-type lectins in *M. chilensis* after exposure to saxitoxin [33,56,57]. On the contrary, these genes were down-regulated in *Argopecten irradians* in response to okadaic acid [32].

One of the most enriched GO terms for the down-regulated DEGs in *A. opercularis* was collagen trimer (Table 8), and collagen triple helix repeat was among the enriched Pfam domains (Table 6). Furthermore, several collagen components form a group of highly connected protein nodes in the network obtained with the down-regulated genes (Figure S2). In *M. galloprovincialis*, after exposure to domoic acid-containing *Pseudo-nitzschia* [31], some collagen genes (7) were down-regulated, although the number of induced genes was greater (13). Collagens are components of the extracellular matrix characterized by the presence of at least one triple-helical domain [66]. They are among the most abundant proteins and have mainly a structural function [66].

Another group of predominantly down-regulated genes (14 up-regulated and 35 down-regulated, File S2) were those coding for heat shock proteins (HSPs). Half of the induced *HSP* genes were mitochondrial forms, and among the repressed ones, the *HSP70* genes predominated. Heat shock proteins are involved in protein folding and can be induced by several types of stress, including high temperature, toxins, pathogens, and hypoxia [67]. Several publications have reported the increased expression of heat shock protein genes in bivalves after exposure to harmful algae toxins [32,57,67,68,69]. Cheng et al. [67] found an expansion of *Hsp70* (heat shock protein 70 kDa) genes from the *Hspa12* subfamily in *Mizuhopecten yessoensis*. Several of these genes were differentially expressed in response to *Alexandrium catenella* exposure (most of them were induced, but there were also some *Hsp70* genes down-regulated [67]). However, Ryan et al. [29] reported the down-regulation of *Hsp68* (a member of the *Hsp70* family) after domoic acid exposure in mouse brain. Furthermore, the exposure to domoic acid-producing *Pseudo-nitzschia* provoked a down-regulation of *HSPs* in *M. galloprovincialis* [31]; these results were coincident with those obtained in the present work.

Another result worth highlighting is that some genes coding for glutamate ionotropic receptors, two genes coding for NMDA receptors, and five coding for kainate (KA) receptors, were all down-regulated in the present study (File S2). The zebrafish *gria2* gene (glutamate ionotropic receptor AMPA 2) was down-regulated after two weeks of low-level domoic acid exposure [24], and the authors suggested that this down-regulation is a compensatory response to elevated glutamatergic activity [24]. It is possible that a similar compensatory mechanism takes place in queen scallops after exposure to domoic acid.

The RT-qPCR results confirm the differential gene expression obtained by RNA-seq. The gene expression changes and expression levels (fold change in relation to control) assessed by the two methods (Figure 5) were very similar. In the determination of gene expression by means of RT-qPCR, the validation of the reference genes for each experimental situation is very important [70,71]. The utilization of RNA-seq expression data allowed us to find more suitable candidate reference genes. Thanks to this, their stability values (Table 10), calculated by geNorm and NormFinder, were low (which means that the expression was stable). The selected reference genes performed better than some traditional reference genes, such as *18S rRNA*, *ACTB*, and *EF1A* [70,71].

## 4. Conclusions

RNA-seq technology was employed to elucidate the transcriptional response triggered by exposure to domoic acid-producing *Pseudo-nitzschia* in the queen scallop *A. opercularis*. A total of 10,144 genes were differentially expressed in the two toxin-exposed groups of scallops in relation to the control group (4913 up-regulated and 5231 down-regulated).

The results obtained are compatible with the hypothesis that exposure to domoic acid-producing *Pseudo-nitzschia* causes oxidative stress in *A. opercularis*. Some consequences of oxidative stress are the impairment of mitochondrial function and oxidation of proteins; therefore, the transcriptional response of the queen scallop tries to counteract these effects with the up-regulation of genes coding for proteins involved in the following: degradation of oxidized proteins (proteasome components), mitochondrial biogenesis (mitochondrial ribosomal proteins, TOMMs, and TIMMs) and antioxidant enzymatic activity (glutathione S-transferases, thioredoxins, glutaredoxins, and copper/zinc superoxide dismutases). The results of the present work and those cited in the literature show that oxidative stress is one of the most common effects of the exposure to toxins and toxin-producing algae, and a part of the harmful effects of the toxins are due to oxidative stress.

A great number of up-regulated genes code for proteins involved in the metabolism of xenobiotics (cytochromes P450, aldo-keto reductases, glutathione S-transferases, and sulfotransferases) and transmembrane transport (solute carriers), while the genes coding for proteins with domains involved in immunological processes (C1q domain, C-type lectins, immunoglobulin domain, fibrinogen beta and gamma chains C-terminal globular domain, and tumor necrosis factors) were mainly down-regulated, with the exception of the C-type lectins.

## 5. Materials and Methods

The methods employed were the same as those previously described [31] except for minor modifications.

### 5.1. Animals

Queen scallops (*A. opercularis*) were obtained from a natural bed in the Ría de Arousa in December 2014 and maintained in a 500-L tank, in the Centro de Investigacións Mariñas, (CIMA, Pedras de Corón, Vilanova de Arousa, Spain), with a continuous unfiltered seawater flow (approximate) of 1200 L/h. On April 9, 2015, 2 random samples of the scallops were obtained, and the remaining scallops (control, group C) were maintained in the tank. The scallops in 1 of the samples (group DB) were analyzed to determine their individual content and concentration of domoic acid. The scallops in the other sample (group DA) were placed in culture baskets and transferred to a raft in the culture area Grove C2 in the Ría de Arousa, where a bloom of *Pseudo-nitzschia* was taking place. The recorded levels of domoic acid in the mussels from that raft showed a maximum of 22 mg DA/kg on April 9 (data obtained from Intecmar, www.intecmar.gal [72]). The scallops were maintained on the raft until April 17, 2015 in order to be exposed to domoic acid-containing *Pseudo-nitzschia*. On that date, the scallops (group DA) were brought back to the laboratory to determine their domoic acid content. The scallops from the control group were sampled on May 12, 2015, after the end of the toxic episode caused by *Pseudo-nitzschia*. From April 9 to May 12, the main characteristics of the seawater, temperature, salinity, light transmission (index of suspended solids), O_2_, and fluorescence (index of phytoplankton abundance) in GROVE and in the area of CIMA were very similar (Figure S3). As previously explained by Pazos et al. [31], the experimental approach (animals naturally exposed to domoic acid-producing *Pseudo-nitzschia*) was chosen because of the difficulty of supplying toxic *Pseudo-nitzschia* under controlled conditions in the laboratory due to the relatively low absorption efficiency of the scallops and to the loss of toxicity of the *Pseudo-nitzschia* cultures.

Digestive glands, gills, and the remaining tissues were obtained by dissecting the scallops. Then, 1 part of each digestive gland was used in the determination of the domoic acid content. The second part was stored in RNAlater (ref. AM7021, Ambion, Life Technologies, Carlsbad, CA, USA) at −80°C until RNA extraction.

### 5.2. Chemicals and Reagents for Toxin Extraction and LC-MS/MS

Methanol for HPLC and formic acid were purchased from RCI Labscan Limited (Bangkok, Thailand) and Sigma-Aldrich (St. Louis, MO, USA), respectively. Ultrapure water was obtained using a Milli-Q Gradient system, coupled with an Elix Advantage 10, both from Millipore (Merck Millipore, Darmstadt, Germany).

### 5.3. Determination of the Domoic Acid Content

To extract the toxin, each digestive gland was placed in aqueous methanol (50%) in a proportion of 1:2 (*w*/*v*) and homogenized with an Ultra-Turrax T25 (IKA, Staufen, Germany). The extract was clarified using centrifugation at 18,000 g at 4 °C for 10 min, retaining a supernatant that was immediately analyzed.

Domoic acid in the obtained extracts was analyzed using LC-MS/MS. The chromatographic separation was carried out using a Thermo Accela chromatographic system (Thermo Fisher Scientific, Waltham, MA, USA), with a high-pressure pump and autosampler. The stationary phase was a solid core Kinetex C18, 50 × 2.1 mm, 2.6 µm particle size, column (Phenomenex, Torrance, CA, USA). An elution gradient, with a flow of 280 µL/min, was used with mobile phase A (formic acid 0.2%) and B (50% MeOH with formic acid 0.2%). The gradient started at 100% A, maintained this condition for 1 min, linearly changed until reaching 55% B in minute 5, was held for 2 min, and then reverted to the initial conditions in order to equilibrate before the next injection. Next, 5 µL of extract, previously filtered through a PES 0.2-µm syringe filter (MFS), were injected.

After the chromatographic separation, domoic acid was detected and quantified by means of a Thermo TSQ Quantum Access MAX triple quadrupole mass spectrometer (Thermo Fisher Scientific, Waltham, MA, USA), equipped with a HESI-II electrospray interface, using positive polarization and SRM mode. The transition 312.18 > 266.18 *m*/*z* was used to quantify the response and 312.18 > 248.18 was used for confirmation. The spectrometer was operated under the following conditions: spray voltage 3400 V, capillary temperature 270 °C, HESI-II temperature 110 °C, sheet gas (nitrogen) 20 (nominal pressure), auxiliary gas (nitrogen) 10 (nominal pressure), collision energy of 15 V, and collision gas (argon) pressure of 1.5 mTorr.

Concentrations of domoic acid were obtained by comparing the response of the quantification transition in the sample extracts with that of a reference solution obtained from NRC Canada. The quantification limit of the method for tissue analysis is less than 20 ng/mL of extract.

### 5.4. RNA Extraction

For digestive gland total RNA isolation, the NucleoSpin RNA kit (ref. 740955, Macherey-Nagel, Düren, Germany) was used following the manufacturer’s protocol. Then, RNA was precipitated with 0.5 volumes of Li CL 7.5 M, and the RNA pellet was dissolved in 50 μL of RNA storage solution (ref. AM7000, Ambion, Life Technologies, Carlsbad, CA, USA). To remove DNA contamination, total RNA was treated with DNA-free (ref. AM1907M, Ambion, Life Technologies, Carlsbad, CA, USA). The integrity and quality of the RNA samples were measured using agarose gel electrophoresis, an Agilent 2100 Bioanalyzer (Agilent Technologies, Santa Clara, CA, USA) and a Nanodrop ND-1000 spectrophotometer (NanoDrop Technologies, Wilmington, DE, USA). The quantity of the total RNA was determined using Qubit 2.0 (Invitrogen, Carlsbad, CA, USA).

### 5.5. Library Preparation and Sequencing

**Figure 6:**
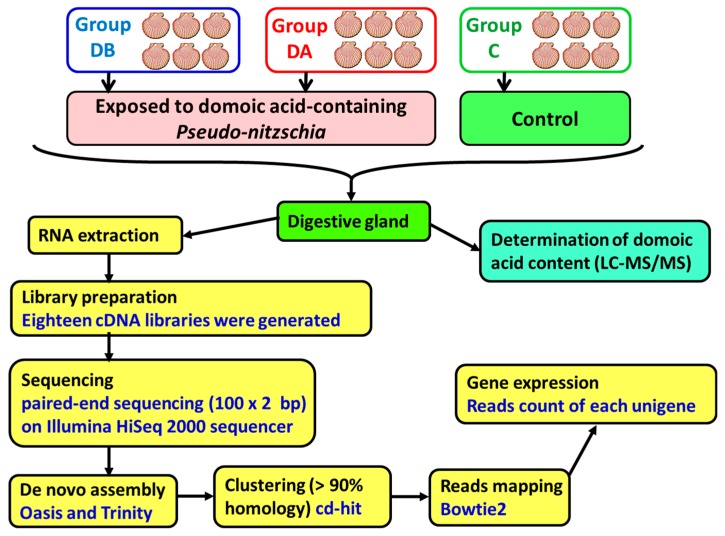
Scheme of the methods employed for sequencing and de novo transcriptome assembly.

A total of 18 cDNA libraries were generated (Figure 6) from the digestive gland of the scallops (6 obtained from each group: DB, DA, and control). The poly(A) + mRNA fraction was isolated from total RNA, and cDNA libraries were obtained following Illumina’s recommendations. Briefly, poly(A) + RNA was isolated on poly-T oligoattached magnetic beads and chemically fragmented prior to reverse transcription and cDNA generation. The cDNA fragments then went through an end repair process, the addition of a single ‘A’ base to the 3’ end, and afterwards, the ligation of the adapters. Finally, the products were purified and enriched with PCR to create the indexed final double-stranded cDNA library. The quality of the libraries was analyzed using a Bioanalyzer 2100 high sensitivity assay; the quantity of the libraries was determined by real-time PCR in a LightCycler 480 (Roche Diagnostics, Mannheim, Germany). Prior to cluster generation in cbot (Illumina), an equimolar pooling of the libraries was performed. The pool of the cDNA libraries was sequenced by paired-end sequencing (100 × 2 bp) on an Illumina HiSeq 2000 sequencer (Illumina, San Diego, CA, USA).

### 5.6. de novo Assembly

Quality control checks of the raw sequencing data were performed with FastQC. The technical adapters were eliminated using Trimgalore software version 0.3.3 (Babraham Bioinformatics, Cambridge, UK) (http://www.bioinformatics.babraham.ac.uk/projects/trim_galore/). Additionally, the reads with a mean Phred score >30 were selected. Subsequently, all the samples were combined, and the complexity of the reads was reduced by removing duplicates. Then, a de novo assembly was performed using the programs Oases, version 0.2.09 [73] and Trinity, version 2.1.1 [74]. The assembled transcripts were clustered (>90% homology) to reduce redundancy using cd-hit software version 4.6. For each sequence, the potential ORFs were detected using Transdecoder software, version 2.0, with standard parameters.

Each sample was then mapped with Bowtie2, version 2.2.6 [75] against the reference transcriptome obtained in the previous step. The good quality reads (Mapping Quality ≥20) were selected to increase the resolution of the count expression. Finally, the expression inference was evaluated by means of the counts of properly paired reads in each transcript.

### 5.7. Differential Expression

The transcriptome expression for each sample was normalized by library size, following the DESeq2 protocols. Considering the whole normalized transcriptome, a study of correlation and Euclidean distance between samples was performed using the statistical software R, version 3.2.3 (www.r-project.org), for identifying possible samples outliers.

**Figure 7:**
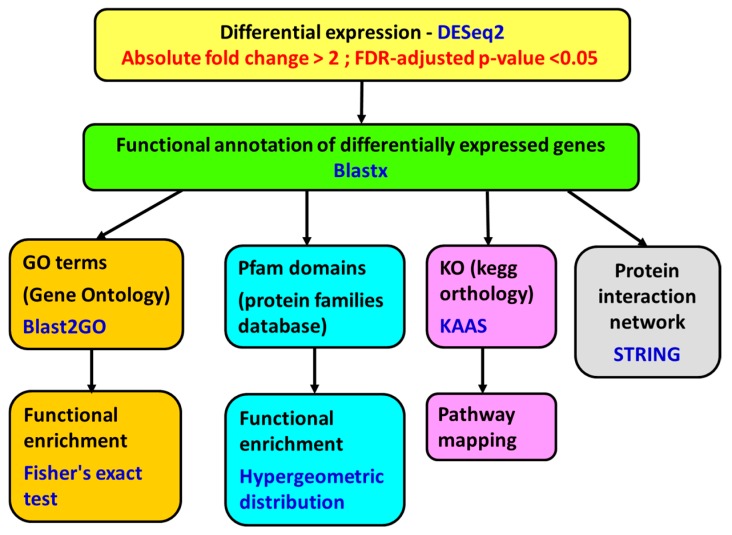
Scheme of the methods employed for differential gene expression analysis, functional annotation, and functional enrichment.

Differential gene expression analysis was performed with DESeq2 algorithm, version 1.8.2 (http://www.bioconductor.org/packages/devel/bioc/html/DESeq2.html). The genes with a fold change of less than −2 or greater than 2 and a *p*-value adjusted using the Benjamini and Hochberg [76] method for controlling false discovery rate (FDR) <0.05 were considered differentially expressed (Figure 7).

A filtering step was performed with the DEGs to remove the transcripts from *Pseudo-nitzschia*; the contigs were blasted against *Mizuhopecten yessoensis* and *Pseudo-nitzschia multistriata* genomes (*E*-value threshold of 10^−10^, word size 12):

ftp://ftp.ncbi.nlm.nih.gov/genomes/all/GCF/002/113/885/GCF_002113885.1_ASM211388v2/GCF_002113885.1_ASM211388v2_genomic.fna.gz

ftp://ftp.ncbi.nlm.nih.gov/genomes/all/GCA/900/005/105/GCA_900005105.1_PsnmuV1.4/GCA_900005105.1_PsnmuV1.4_genomic.fna.gz

The contigs that had a lower *E*-value versus *Pseudo-nitzschia* compared with *M. yessoensis* were discarded (approximately 0.7% of the sequences).

### 5.8. Functional Annotation

The genes were annotated using Blastx [77] against the Uniprot database and Blastn [77] against the NCBI nucleotide database (*E*-value threshold of 10^−2^). Then, the annotation was expanded by incorporating information from the species, gene name, and functions using gene ontology and protein structure domains associated with the transcript using InterPro (https://www.ebi.ac.uk/interpro/). The genes were also annotated with Blast2GO software version 4.1.9 (BioBam Bioinformatics S.L, Valencia, Spain) [78,79], using local Blastx 2.4.0+ against a database of *Mizuhopecten yessoensis* and *Crassostrea gigas* proteins obtained from NCBI (*E*-value threshold of 10^−3^):

ftp://ftp.ncbi.nlm.nih.gov/genomes/Mizuhopecten_yessoensis/protein/protein.fa.gz (last modification 19/06/2017)

ftp://ftp.ncbi.nlm.nih.gov/genomes/Crassostrea_gigas/protein/protein.fa.gz (last modification 06/02/2017)

Orthologue assignment and pathway mapping were performed on the KEGG Automatic Annotation Server (KAAS, [80]) using Blast and the bi-directional best hit (BBH) method (http://www.genome.jp/tools/kaas/).

### 5.9. Functional Enrichment

A functional enrichment study was performed using the Pfam [81] functional information. This study is based on hypergeometric distribution [82] using the statistical software R version 3.2.3 (www.r-project.org). The differentially expressed genes were also subjected to GO enrichment analysis with Blast2GO version 4.1.9. (BioBam Bioinformatics S.L, Valencia, Spain) using Fisher’s exact test [83] (up- and down-regulated genes were analyzed separately). The false discovery rate (FDR) adjusted p-value [76] was set at a cutoff of 0.05.

### 5.10. Protein Network Analysis

To search for the protein–protein interactions, network analyses using the String 10.5 algorithm [84] were performed. The putative human homologues of proteins coded by the up-regulated and the down-regulated genes in the *A. opercularis* digestive gland were identified by means of a Blastx search [85] against the STRING human protein database (9606.protein.sequences.v10.fa), with an E-value threshold of 10^−5^. The top Blastx search results were used as input in the String program. The up-regulated and the down-regulated genes were analyzed separately.

### 5.11. Real Time RT-qPCR Validation

cDNA was synthesized from 0.5 μg of total RNA with the iScript™cDNA Synthesis kit (ref. 170-8891, BioRad, Hercules, CA, USA) in a 20-µL reaction volume, and the conditions were 5 min at 25 °C, 30 min at 42 °C, and 5 min at 85 °C.

For the relative quantification of gene expression by means of RT-qPCR, a normalization step must be performed using internal reference genes, whose expression levels are stable [38,86,87,88]. Suitable reference genes should be selected for each experimental condition to ensure their stable expression [70,89].

A total of 6 reference gene candidates (Table 9), *VAMP7*, *RPS4*, *MYH9*, *EIF4EBP2*, *DNAJ*, and *RAP1B* and 5 target genes (Table 9), *CYP2C14*, *SLC16A12*, *ANT1*, *SLC16A13*, and *SLC6A9*, were used in the gene expression study. The candidate reference genes were selected for their stable expression based on the RNA-seq data. Oligonucleotide primers were designed with OligoAnalyzer 3.1 (http://eu.idtdna.com/analyzer/Applications/OligoAnalyzer/; Integrated DNA Technologies, Leuven, Belgium) from the sequences in Table 9 and were synthesized by Integrated DNA Technologies (Leuven, Belgium). The primer sequences and amplicon lengths are listed in Table 9. The specificity of the primers was confirmed by the presence of a single peak in the melting curve and by the presence of a single band of the expected size when PCR products were run in a 2% agarose gel. The PCR amplification efficiency (E) of each transcript was determined by means of Real-Time PCR Miner software (Version 4.0; http://www.miner.ewindup.info/ [90]). The mean amplification efficiency (E) of each amplicon (Table 9) was used in the calculation of gene expression.

Real-time qPCR analysis was conducted in technical duplicates and 6 biological replicates, in 96-well reaction plates on an iCycler iQ^®^ Real-Time System (Bio-Rad, Hercules, CA, USA, 2003). The PCR final volume was 20 μL, containing 4 μL of 1:5 diluted cDNA (20 ng of cDNA), 10 μL of SsoFast EvaGreen Supermix (ref. 172-5201, Bio-Rad, Hercules, CA, USA), 400 nM of forward and reverse primers, and 4.4 μL of PCR-grade water. The cycling conditions were as follows: 30 s at 95 °C (initial template denaturation) and 40 cycles of 5 s at 95 °C (denaturation) followed by 10 s at 60 °C (annealing and elongation) and 10 s at 75 °C for fluorescence measurement. At the end of each run, a melting curve was carried out: 95 °C for 20 s and 60 °C for 20 s followed by an increase in temperature from 60 to 100 °C (with temperature increases in steps of 0.5 °C every 10 s). Baseline values were automatically determined for all the plates using Bio-Rad iCycler iQ software V3.1 (IQ™ Real-Time PCR Detection System). The threshold value was set manually at 100 RFU (relative fluorescence units) to calculate the Cq values. Non-reverse transcriptase controls and non-template controls (NTC) were also included in each run.

The gene expression was normalized to reference genes that had stable expression levels [38,86,87,88]. The gene expression stability of candidate reference genes was analyzed using 3 Microsoft Excel-based software applications, geNorm V3.5 [38], NormFinder V0.953 [86], and BestKeeper V1 [88]. The non-normalized expression (Q) was calculated using the equation Q = (1 + E)^–Cq^. Then, the expression was normalized by dividing it by the normalization factor (the geometric mean of the non-normalized expression of the selected reference genes) [89].

The statistical analyses were performed with the IBM SPSS Statistics 24.0 package (IBM SPSS, Chicago, IL, USA). The data were tested for normality (Shapiro–Wilk test) and for homogeneity of variance (Levene’s test). The gene expression was log-transformed (base 2) to meet the requirements of normality and homogeneity of variances. The expression of target genes in domoic acid-exposed scallops (groups DB and DA) in relation to the control group was compared using ANOVA and post hoc Dunnett’s *t* test. *p* <0.05 was considered statistically significant.

## Figures and Tables

**Table 1:** Domoic acid concentration (ng/g digestive gland wet weight), wet weight (g) of the soft tissues (Total weight), and wet weight (g) of the digestive gland (DG weight) in sampled scallops (*Aequipecten opercularis*).

Group	Sampling Date	Domoic Acid (ng/g)		Total Weight (g)		DG Weight (g)	
		Mean	SD	Mean	SD	Mean	SD
DB	09/04/2015	1361	803.8	3.663	0.739	0.218	0.053
DA	17/04/2015	6680	1611.4	5.102	0.328	0.509	0.076
Control (C)	12/05/2015	BLOQ ^1^	BLOQ ^1^	2.314	0.489	0.156	0.149

^1^ BLOQ: below the limit of quantification; SD: standard deviation.

**Table 2:** Summary of Illumina transcriptome sequencing and assembly for *A. opercularis* digestive glands.

**Summary of Raw Reads Data:**	
Total number of filtered reads	968,035,762
Average read length alter filtering (bp)	100
Sequence quality ≥ Q30 (%)	95
Mean quality score	38
GC%	39
**Summary of the Assembled Transcriptome**	
Number of assembled unigenes	142,137
Contig N50 Length (bp)	1845
Minimum contig length (bp)	200
Maximum contig length (bp)	17,867
Average contig length (bp)	1343.9
Total length in contigs (bp)	191,023,300

**Table 3:** The top 25 up-regulated genes classified by false discovery rate (FDR)-adjusted p-value (padj) of group DA.

Sequence ID	Description	FC DB	padj DB	FC DA	padj DA
ci|000048247|Bact|Sample_DA|2	fatty acid-binding protein, brain-like	4.02	5.18 × 10^−13^	17.42	3 × 10^−5^
ci|000123653|Bact|Sample_DB|2	ganglioside-induced differentiation-associated protein 1-like	2.43	1.52 × 10^−5^	4.35	2.18 × 10^−71^
ci|000014617|Bact|Sample_DA|2	fatty acid-binding protein homolog 5 isoform X2	8.41	1.20 × 10^−13^	35.70	1.46 × 10^−54^
ci|000041038|Bact|Sample_C|2	fatty acid-binding protein, brain-like	3.82	4.54 × 10^−8^	18.21	8.69 × 10^−54^
ci|000053956|Bact|Sample_C|2	fatty acid-binding protein, brain-like	3.83	4.61 × 10^−9^	17.75	1.17 × 10^−53^
ci|000005112|Bact|Sample_DB|2	fatty acid-binding protein homolog 5 isoform X2	6.09	8.48 × 10^−13^	26.48	1.16 × 10^−49^
ci|000017145|Bact|Sample_C|2	selenoprotein F-like	2.01	4.34 × 10^−11^	2.75	5.18 × 10^−47^
ci|000005129|Bact|Sample_C|2	uncharacterized protein LOC110453031	2.11	9.79 × 10^−13^	3.40	1.02 × 10^−45^
ci|000000144|Bact|Sample_DA|2	fatty acid-binding protein, brain-like	4.29	1.10 × 10^−11^	18.11	1.02 × 10^−45^
ci|000005171|Bact|Sample_DB|2	---NA---	6.89	1.43 × 10^−9^	26.37	2.23 × 10^−42^
ci|000069997|Bact|Sample_DA|2	acylpyruvase FAHD1, mitochondrial	2.52	3.20 × 10^−8^	5.13	1.87 × 10^−41^
ci|000047776|Bact|Sample_C|2	sulfotransferase family cytosolic 1B member 1-like	3.33	1.24 × 10^−5^	13.03	2.22 × 10^−41^
ci|000033268|Bact|Sample_DB|2	arylsulfatase B-like	8.26	3.74 × 10^−26^	11.50	6.15 × 10^−41^
ci|000026813|Bact|Sample_DA|2	sulfotransferase family cytosolic 1B member 1-like isoform X1	5.10	5.74 × 10^−15^	14.18	2.34 × 10^−39^
ci|000049206|Bact|Sample_C|2	uncharacterized protein LOC110453031	2.10	6.28 × 10^−11^	3.41	2.54 × 10^−38^
ci|000050101|Bact|Sample_DA|2	fatty acid-binding protein, brain-like	5.64	2.58 × 10^−7^	29.68	3.39 × 10^−38^
ci|000056604|Bact|Sample_DA|2	sulfotransferase family cytosolic 1B member 1-like	5.87	2.29 × 10^−15^	15.32	3.51 × 10^−38^
ci|000027873|Bact|Sample_DB|2	cytochrome b5-like	2.12	5.81 × 10^−8^	3.19	2.28 × 10^−36^
ci|000039930|Bact|Sample_DB|2	sulfotransferase family cytosolic 1B member 1-like	7.09	9.44 × 10^−22^	15.69	3.39 × 10^−36^
ci|000065147|Bact|Sample_DB|2	fatty acid-binding protein homolog 5 isoform X2	4.36	1.92 × 10^−6^	19.45	2.59 × 10^−35^
ci|000006862|Bact|Sample_DA|2	fatty acid-binding protein, brain-like	4.92	1.72 × 10^−6^	28.74	1.03 × 10^−34^
ci|000020752|Bact|Sample_DB|2	---NA---	8.09	3.57 × 10^−32^	7.28	1.07 × 10^−34^
ci|000016532|Bact|Sample_DA|2	fatty acid-binding protein, brain-like	5.97	2.32 × 10^−8^	30.92	1.21 × 10^−34^
ci|000059056|Bact|Sample_DA|2	dimethylaniline monooxygenase [N-oxide-forming] 5-like isoform X1	4.07	7.94 × 10^−10^	8.29	1.43 × 10^−34^
ci|000093179|Bact|Sample_DB|2	uncharacterized protein LOC110453031	2.67	2.16 × 10^−13^	4.31	2.33 × 10^−33^

FC: fold change.

**Table 4:** The top 25 down-regulated genes classified by FDR-adjusted p-value (padj) of group DA.

Sequence ID	Description	FC DB	padj DB	FC DA	padj DA
ci|000106611|Bact|Sample_C|2	---NA---	−57.59	1.68 × 10^−42^	−139.49	6.68 × 10^−64^
ci|000007989|Bact|Sample_C|2	F-box only protein 33	−7.31	2.69 × 10^−11^	−72.56	6.79 × 10^−58^
ci|000015465|Bact|Sample_C|2	---NA---	−327.41	8.87 × 10^−67^	−438.98	6.31 × 10^−54^
ci|000021112|Bact|Sample_C|2	---NA---	−77.85	2.14 × 10^−60^	−317.56	7.15 × 10^−48^
ci|000005274|Bact|Sample_C|2	---NA---	−371.71	5.02 × 10^−52^	−372.69	2.17 × 10^−45^
ci|000028047|Bact|Sample_C|2	---NA---	−363.88	3.57 × 10^−56^	−393.01	4.76 × 10^−45^
ci|000101976|Bact|Sample_C|2	---NA---	−296.03	4.63 × 10^−36^	−314.75	3.8 × 10^−44^
ci|000000908|Bact|Sample_C|2	---NA---	−28.16	1.34 × 10^−23^	−107.94	4.64 × 10^−44^
ci|000036200|Bact|Sample_DB|2	probable serine/threonine-protein kinase kinX	−2.76	6.84 × 10^−6^	−10.59	5.31 × 10^−43^
ci|000063999|Bact|Sample_C|2	---NA---	−544.00	1.59 × 10^−52^	−705.51	5.33 × 10^−43^
ci|000012018|Bact|Sample_DB|2	zwei Ig domain protein zig-2-like	−6.84	2.58 × 10^−13^	−42.34	1.91 × 10^−42^
ci|000012910|Bact|Sample_C|2	---NA---	−19.42	2.97 × 10^−35^	−199.53	2.71 × 10^−42^
ci|000023297|Bact|Sample_C|2	---NA---	−79.85	9.38 × 10^−54^	−322.38	4.58 × 10^−42^
ci|000038123|Bact|Sample_C|2	---NA---	−80.83	4.63 × 10^−47^	−266.21	9.28 × 10^−42^
ci|000020939|Bact|Sample_C|2	---NA---	−2.05	3.09 × 10^−2^	−58.46	1.05 × 10^−39^
ci|000005202|Bact|Sample_C|2	---NA---	−315.71	5.05 × 10^−46^	−181.22	4.46 × 10^−37^
ci|000039871|Bact|Sample_C|2	neuroglian-like isoform X1	−7.48	3.70 × 10^−11^	−44.27	1.65 × 10^−35^
ci|000013710|Bact|Sample_C|2	---NA---	−404.07	4.63 × 10^−47^	−401.37	1.99 × 10^−35^
ci|000012324|Bact|Sample_C|2	---NA---	−163.57	5.05 × 10^−46^	−373.00	1.47 × 10^−34^
ci|000023304|Bact|Sample_DB|2	gliomedin-like isoform X4	−2.42	2.42 × 10^−3^	−5.65	3.18 × 10^−33^
ci|000051151|Bact|Sample_C|2	---NA---	−56.53	1.13 × 10^−30^	−221.69	1.45 × 10^−32^
ci|000088531|Bact|Sample_C|2	---NA---	−55.44	9.80 × 10^−33^	−124.60	3.09 × 10^−32^
ci|000072709|Bact|Sample_C|2	---NA---	−293.26	3.06 × 10^−39^	−243.75	7.51 × 10^−32^
ci|000096728|Bact|Sample_C|2	uncharacterized protein LOC110458629	−9.55	9.51 × 10^−18^	−20.79	7.51 × 10^−32^
ci|000074174|Bact|Sample_C|2	---NA---	−33.35	1.02 × 10^−23^	−71.61	2.96 × 10^−31^

FC: fold change.

**Table 5:** Summary of the functional annotation results.

Functional Annotation	Number	%
**Differentially expressed unigenes**	**10,144**	**100**
With Blastx hit	6081	59.95
With GO terms	3451	34.02
With enzyme code	638	6.29
With KO orthologue	1728	17.03
With PFAM domains	4379	43.17
**All unigenes**	**142,137**	**100**
With Blastx hit	67,925	47.79
With GO terms	38,825	27.32
With enzyme code	6991	4.92
With KO orthologue	13,978	9.83
With PFAM domains	46,664	32.83

**Table 6:** The top twenty Pfam families that were significantly (FDR-adjusted *p*-value <0.05) enriched.

Pfam	Description	N° Genes	UP/DOWN	padj FDR
PF00386.16	C1q domain	13	DOWN	2.08 × 10^−137^
PF00059.16	Lectin C-type	27	UP	4.77 × 10^−66^
PF14259.1	RNA recognition motif. (RRM_6)	28	UP	8.64 × 10^−54^
PF00076.17	RNA recognition motif. (RRM_1	37	UP	5.38 × 10^−51^
PF07690.11	Major Facilitator Superfamily	34	UP	5.18 × 10^−46^
PF13927.1	Immunoglobulin domain	55	DOWN	7.61 × 10^−46^
PF13893.1	RNA recognition motif. (RRM_5)	22	UP	9.46 × 10^−44^
PF13414.1	tetratricopeptide repeat	18	DOWN	2.98 × 10^−38^
PF00067.17	Cytochrome P450	45	UP	4.06 × 10^−38^
PF01391.13	Collagen triple helix repeat (20 copies)	29	DOWN	6.16 × 10^−36^
PF12695.2	Alpha/beta hydrolase fold	10	DOWN	2.15 × 10^−35^
PF00400.27	WD domain, G-beta repeat;	20	UP	9.61 × 10^−35^
PF05721.8	Phytanoyl-CoA dioxygenase (PhyH)	9	UP	1.01 × 10^−34^
PF12697.2	Alpha/beta hydrolase fold	6	DOWN	1.07 × 10^−34^
PF13424.1	tetratricopeptide repeat	14	DOWN	1.72 × 10^−34^
PF00009.22	Elongation factor Tu GTP binding domain	23	UP	5.23 × 10^−32^
PF00515.23	tetratricopeptide repeat	16	DOWN	1.52 × 10^−31^
PF00531.17	Death domain	18	DOWN	5.62 × 10^−31^
PF13181.1	tetratricopeptide repeat	10	DOWN	6.11 × 10^−31^
PF07719.12	tetratricopeptide repeat	19	DOWN	7.32 × 10^−30^

UP/DOWN indicates if most of the genes in the category were up- or down-regulated.

**Table 7:** The top 10 enriched gene ontology (GO) terms (classified by FDR) for the up-regulated genes in biological process (BP), molecular function (MF), and cellular component (CC) categories.

GO ID	GO Name	GO Type	padj FDR	Fold Enrichment
GO:0008152	metabolic process	BP	8.55 × 10^−165^	2.38
GO:0055114	oxidation-reduction process	BP	3.42 × 10^−120^	4.21
GO:1901575	organic substance catabolic process	BP	8.52 × 10^−60^	4.89
GO:0044237	cellular metabolic process	BP	3.81 × 10^−58^	2.00
GO:0009056	catabolic process	BP	1.89 × 10^−54^	4.46
GO:0044248	cellular catabolic process	BP	3.27 × 10^−53^	4.74
GO:0071704	organic substance metabolic process	BP	9.09 × 10^−53^	1.86
GO:0044238	primary metabolic process	BP	7.05 × 10^−49^	1.85
GO:0019752	carboxylic acid metabolic process	BP	1.18 × 10^−47^	3.66
GO:0043436	oxoacid metabolic process	BP	2.22 × 10^−47^	3.64
GO:0003824	catalytic activity	MF	1.39 × 10^−189^	2.53
GO:0016491	oxidoreductase activity	MF	2.06 × 10^−134^	4.33
GO:0070003	threonine-type peptidase activity	MF	1.44 × 10^−71^	72.62
GO:0004298	threonine-type endopeptidase activity	MF	1.44 × 10^−71^	72.62
GO:0016616	oxidoreductase activity, acting on the CH-OH group of donors, NAD or NADP as acceptor	MF	1.21 × 10^−48^	14.26
GO:0016614	oxidoreductase activity, acting on CH-OH group of donors	MF	1.13 × 10^−44^	9.75
GO:0048037	cofactor binding	MF	8.82 × 10^−42^	3.53
GO:0050662	coenzyme binding	MF	6.23 × 10^−39^	4.61
GO:0004576	oligosaccharyl transferase activity	MF	3.37 × 10^−28^	111.72
GO:0016782	transferase activity, transferring sulfur-containing groups	MF	4.78 × 10^−26^	6.61
GO:0005737	Cytoplasm	CC	2.87 × 10^−100^	3.60
GO:0000502	proteasome complex	CC	6.28 × 10^−82^	30.23
GO:1905369	endopeptidase complex	CC	6.28 × 10^−82^	30.23
GO:1905368	peptidase complex	CC	4.48 × 10^−78^	25.22
GO:0044424	intracellular part	CC	2.84 × 10^−75^	2.37
GO:0005839	proteasome core complex	CC	1.44 × 10^−71^	72.62
GO:0044444	cytoplasmic part	CC	4.88 × 10^−68^	3.45
GO:0005622	intracellular	CC	2.16 × 10^−67^	2.22
GO:0044464	cell part	CC	1.94 × 10^−63^	2.15
GO:0005623	Cell	CC	2.00 × 10^−62^	2.13

**Table 8:** The top 10 enriched gene ontology (GO) terms (classified by FDR) for the down-regulated genes in biological process (BP), molecular function (MF), and cellular component (CC) categories.

GO ID	GO Name	GO Type	padj FDR	Fold Enrichment
GO:0006836	neurotransmitter transport	BP	4.01 × 10^−34^	17.69
GO:0050794	regulation of cellular process	BP	7.32 × 10^−27^	1.98
GO:0007154	cell communication	BP	7.32 × 10^−27^	2.45
GO:0023052	Signaling	BP	7.07 × 10^−26^	2.42
GO:0050789	regulation of biological process	BP	2.48 × 10^−25^	1.93
GO:0007165	signal transduction	BP	3.18 × 10^−24^	2.38
GO:0065007	biological regulation	BP	1.39 × 10^−18^	1.73
GO:0006468	protein phosphorylation	BP	5.33 × 10^−18^	3.02
GO:0050896	response to stimulus	BP	3.98 × 10^−17^	1.92
GO:0051716	cellular response to stimulus	BP	1.35 × 10^−16^	1.98
GO:0005326	neurotransmitter transporter activity	MF	9.58 × 10^−37^	21.66
GO:0005328	neurotransmitter/sodium symporter activity	MF	9.58 × 10^−37^	21.66
GO:0015370	solute/sodium symporter activity	MF	9.58 × 10^−37^	21.66
GO:0015294	solute/cation symporter activity	MF	4.01 × 10^−34^	17.69
GO:0015081	sodium ion transmembrane transporter activity	MF	4.01 × 10^−34^	17.69
GO:0015293	symporter activity	MF	2.50 × 10^−31^	14.61
GO:0046873	metal ion transmembrane transporter activity	MF	6.68 × 10^−30^	8.87
GO:0015291	secondary active transmembrane transporter activity	MF	1.18 × 10^−23^	8.72
GO:0003700	DNA-binding transcription factor activity	MF	6.43 × 10^−23^	4.26
GO:0015077	monovalent inorganic cation transmembrane transporter activity	MF	2.62 × 10^−20^	5.77
GO:0005667	transcription factor complex	CC	3.83 × 10^−17^	3.38
GO:0005581	collagen trimer	CC	2.61 × 10^−13^	11.58
GO:0005856	cytoskeleton	CC	2.94 × 10^−8^	2.24
GO:0099513	polymeric cytoskeletal fiber	CC	8.14 × 10^−8^	3.24
GO:0005874	microtubule	CC	1.18 × 10^−7^	3.32
GO:0099080	supramolecular complex	CC	2.40 × 10^−7^	3.10
GO:0099081	supramolecular polymer	CC	2.40 × 10^−7^	3.10
GO:0099512	supramolecular fiber	CC	2.40 × 10^−7^	3.10
GO:0030286	dynein complex	CC	4.68 × 10^−7^	4.58
GO:0034703	cation channel complex	CC	1.51 × 10^−6^	19.63

**Table 9:** Genes selected for RT-qPCR: sequence names, description, gene symbols, primers, and amplicon length (bp) for each primer pair and average efficiency (E).

Sequence ID	Description	Symbol	Sense Primer	Antisense Primer	bp	E
ci|000063635|Bact|Sample_C|2	vesicle-associated membrane protein 7-like	*VAMP7*	ACTGACAATCGTAGTGGTGCTG	GCAGTGGTGGTGGTAGTTGATG	84	0.8584
ci|000050253|Bact|Sample_C|2	40S ribosomal protein S4	*RPS4*	AATGGGTTACCGAGGACG	CACCACTCAGTTTGTCCAAC	80	0.7934
ci|000036898|Bact|Sample_DA|2	myosin heavy chain, non-muscle isoform X11-MYOSIN 9	*MYH9*	CGCCATTACAGATGCAGCA	GATTCACCTGTGCAGAGG	75	0.8267
ci|000071167|Bact|Sample_DA|2	eukaryotic translation initiation factor 4E-binding protein 2-like	*EIF4EBP2*	CCAGGAGTAACAGCACCAG	TGTCCATCTCGAACTGTGG	130	0.8659
ci|000071620|Bact|Sample_C|2	molecular chaperone DNAJ/HSP40	*DNAJ*	GCCTATGATAATGCCTCTACG	CTAGGACGTGTGACATATTCC	110	0.8501
ci|000093955|Bact|Sample_DB|2	ras-related protein Rap-1b isoform 1 precursor	*RAP1B*	TGAAGTGGATGGACAACAGTG	TGTGCTGTGATGGAATACACC	129	0.8648
ci|000058258|Bact|Sample_DA|2	cytochrome p450 2c14-like isoform x2	*CYP2C14*	GCCTGGTCCTTCTGGATAC	CTTCAAGCTGAATACGTCACC	115	0.8697
ci|000104366|Bact|Sample_C|2	sodium- and chloride-dependent glycine transporter 1-like	*SLC6A9*	TTCTGAGTCGAATAGCTCTGG	TATCAACCACGGTCGTCTC	80	0.8500
ci|000028690|Bact|Sample_DA|2	monocarboxylate transporter 12-like	*SLC16A12*	CCTGCTATGATTGCTTACGG	CAGTCCAACATCGCTACAG	83	0.9682
ci|000032679|Bact|Sample_DA|2	amino acid transporter antl1-like isoform x1	*ANT1*	AAGCTGGCAGATATACAGTG	TTGGTGTTCCGAACCAGG	189	0.8906
ci|000000293|Bact|Sample_DB|2	monocarboxylate transporter 13-like isoform x1	*SLC16A13*	AAGACATCCAGCCATGAGTTG	CTTCCAAGAACAACGAACCAG	86	0.8463

**Table 10:** Rank of the six candidate reference genes in quantitative real-time reverse transcription–polymerase chain reaction (RT–qPCR), calculated by geNorm, NormFinder, and BestKeeper analysis.

Rank	GeNorm (Average M)		Normfinder (Stability)		BestKeeper (*r*)		BestKeeper (SD)	
1	*EIF4EBP2-RAP1B*	0.399	*EIF4EBP2*	0.137	*RPS4*	0.92	*DNAJ*	0.53
2	*EIF4EBP2-RAP1B*	0.399	*RPS4*	0.151	*EIF4EBP2*	0.9	*VAMP7*	0.54
3	*RPS4*	0.431	*VAMP7*	0.163	*RAP1B*	0.86	*RPS4*	0.55
4	*VAMP7*	0.487	*RAP1B*	0.192	*VAMP7*	0.79	*EIF4EBP2*	0.55
5	*DNAJ*	0.504	*DNAJ*	0.216	*MYH9*	0.77	*MYH9*	0.63
6	*MYH9*	0.584	*MYH9*	0.237	*DNAJ*	0.68	*RAP1B*	0.69

## Supplementary Materials

The following are available online at http://www.mdpi.com/2072-6651/11/2/97/s1, Table S1: Wet weight and domoic acid content of the queen scallops (*A. opercularis*) in the three groups of the study; Table S2: List of level-2 enriched gene ontology (GO) terms for differentially expressed genes in biological process (BP), molecular function (MF), and cellular component (CC) categories; Figure S1: Network showing interactions (confidence view) of proteins coded by genes up-regulated in the present study; Figure S2: Network showing interactions (confidence view) of proteins coded by genes down-regulated in the present study; Figure S3: Fluorescence (relative units), dissolved oxygen (mL/L), salinity, temperature (°C), and light transmission (%) of the seawater between 1- and 5-m depth between April 9 and May 12, in the area of CIMA (laboratory), and GROVE (transplanted scallops); File S1: Nucleotide sequences of differentially expressed genes (in fasta format); File S2: List of differentially expressed genes in groups DA and DB (in relation to the control group); File S3: Significantly enriched Pfam families among the differentially expressed genes; File S4: Significantly enriched GO terms; File S5: List of KO (KEGG Orthologues) for the differentially expressed genes and for all the genes; File S6: Results of a Blastx search of up-regulated genes against the STRING human protein database (9606.protein.sequences.v10.fa), and list of input proteins in STRING network analysis; File S7: Results of a Blastx search of down-regulated genes against the STRING human protein database (9606.protein.sequences.v10.fa), and list of input proteins in STRING network analysis; and File S8: List of the differentially expressed genes belonging to the solute carriers (*SLC*) superfamily.
