# Supplementary material for: RNA-Seq Transcriptome Profiling of the Queen Scallop (Aequipecten opercularis) Digestive Gland after Exposure to Domoic Acid-Producing Pseudo-nitzschia
Source: Toxins (Basel). 2019 Feb 6;11(2):97. doi: 10.3390/toxins11020097 (PMC6410316; doi:10.3390/toxins11020097)
Supplement: Supplementary file 1 [file toxins-11-00097-s001.zip › toxins-426382-english-supplementary (Tables and Figures).docx]

Supplementary Materials: RNA-Seq Transcriptome Profiling of the Queen Scallop (*Aequipecten opercularis*) Digestive Gland after Exposure to Domoic Acid-Producing *Pseudo-nitzschia*

Pablo Ventoso, Antonio J. Pazos *, M. Luz Pérez-Parallé, Juan Blanco, Juan C. Triviño
and José L. Sánchez


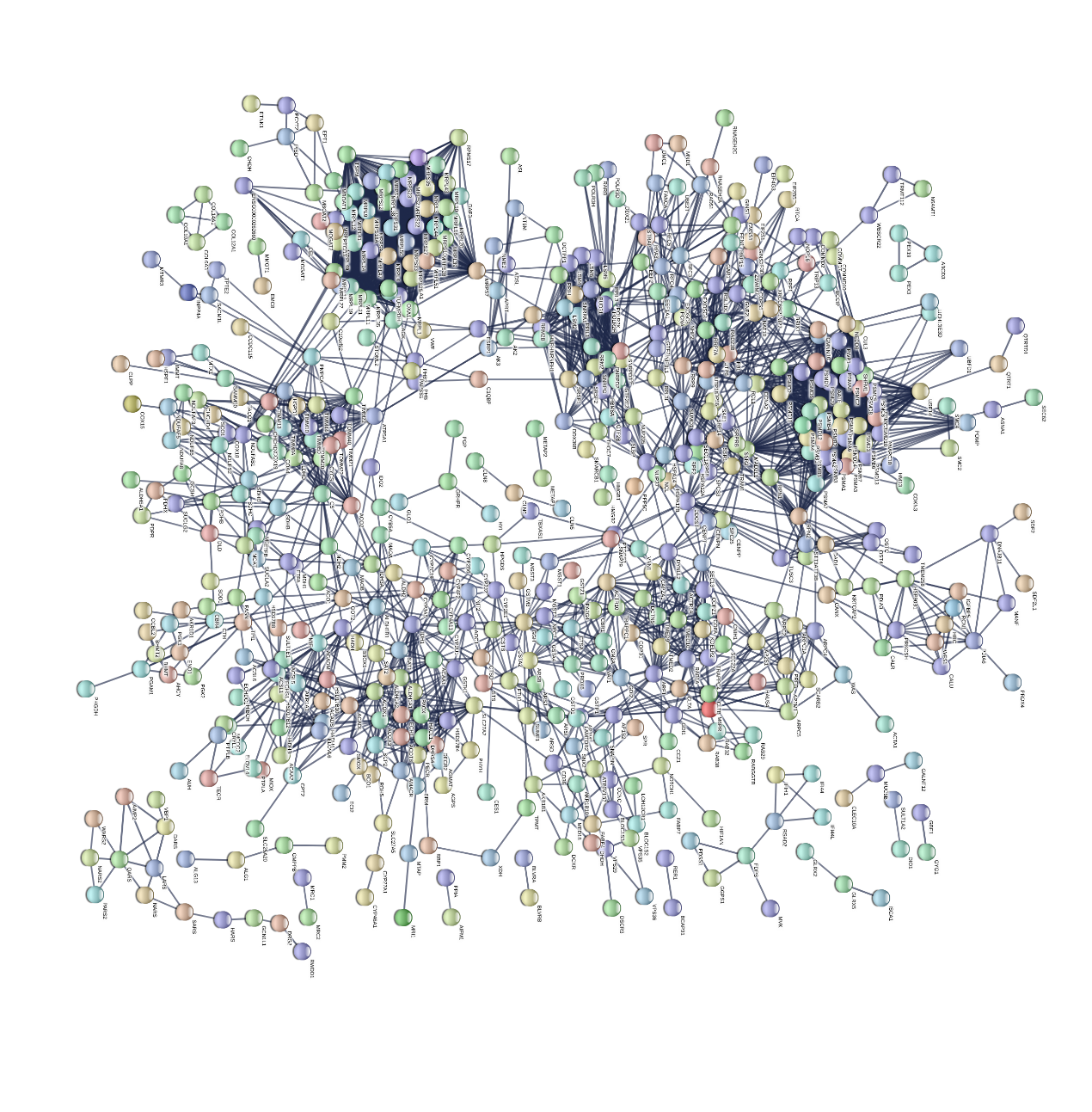


**Figure S1.** Network showing interactions (confidence view) of proteins coded by genes up-regulated in the present study. Network was constructed using the String 10.5 algorithm and obtained in the highest confidence (0.9) mode. Proteins were named according to the human protein name (the full list of names is available in File S6).


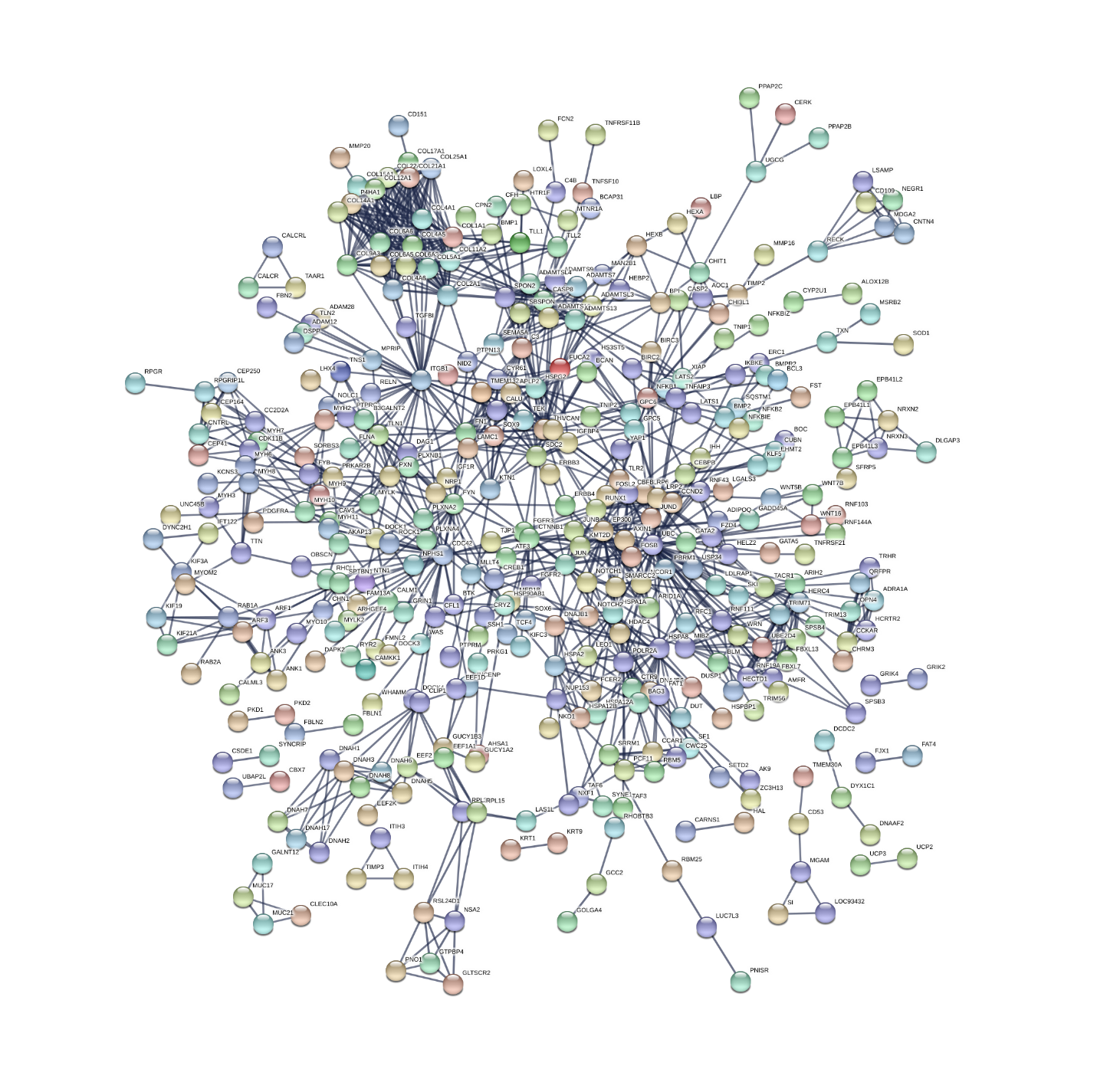


**Figure S2.** Network showing interactions (confidence view) of proteins coded by genes down-regulated in the present study. Network was constructed using the String 10.5 algorithm and obtained in the highest confidence (0.9) mode. Proteins were named according to the human protein name (the full list of names is available in File S7).


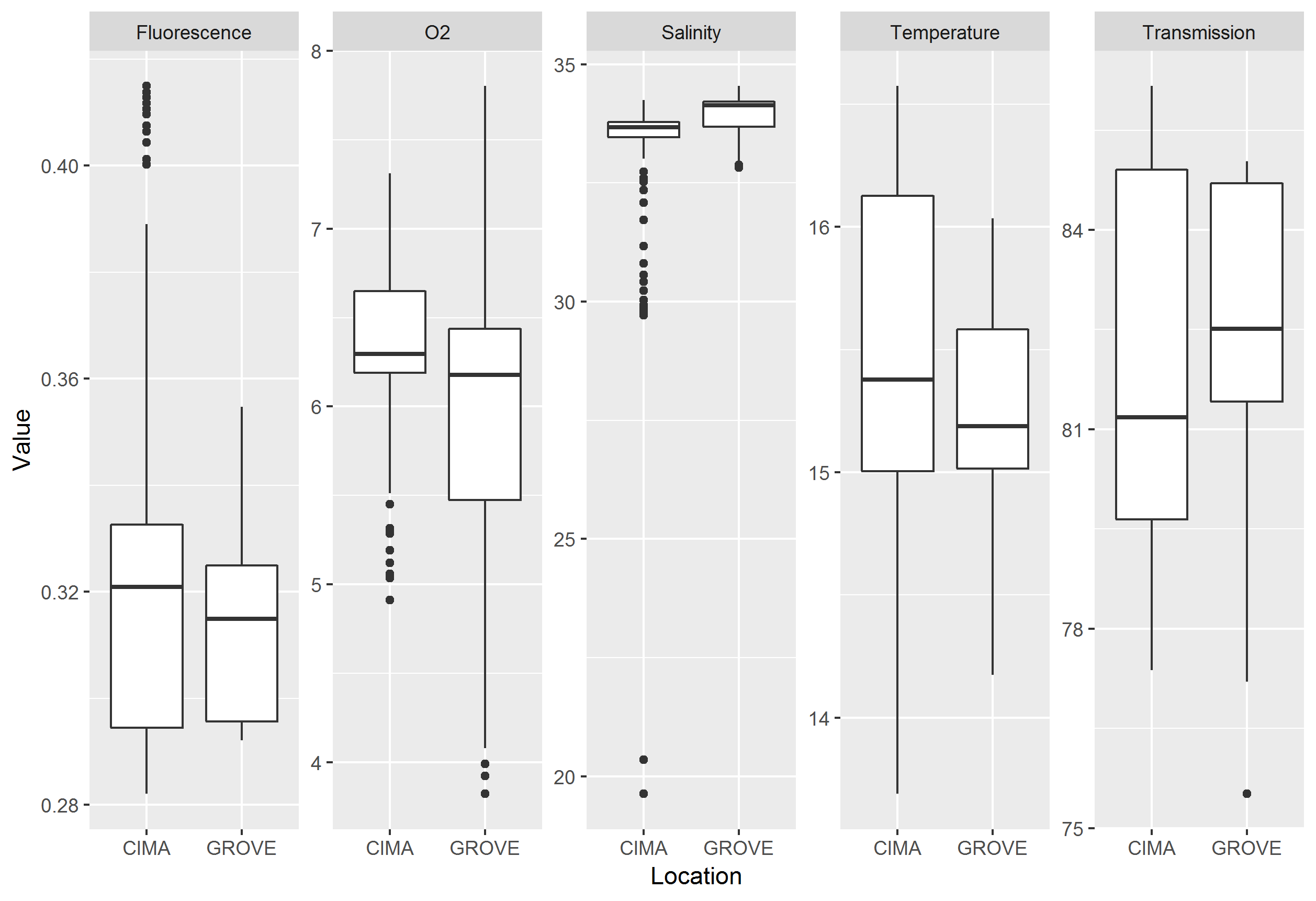


**Figure S3.** Fluorescence (relative units), dissolved oxygen (mL/L), salinity, temperature (°C) and light transmission (%) of the seawater between 1 and 5 m depth, between 9 April and 12 May, in the area of CIMA (laboratory), and GROVE (transplanted scallops).

**Table S1.** Wet weight and domoic acid content of the queen scallops (A. opercularis) in the three groups of the study. Wet weight (g) of the soft tissues (Total weight), wet weight (g) of the digestive gland (DG weight) and domoic acid concentration (ng/g digestive gland wet weight) in sampled scallops are shown.

| **Sampling Date** | **Sample** | **Total Weight (g)** | **DG Weight (g)** | **Domoic Acid (ng/g)** |
| --- | --- | --- | --- | --- |
| 09/04/2015 | DB1 | 3.6875 | 0.2316 | 2539 |
| 09/04/2015 | DB2 | 3.5444 | 0.2918 | 527 |
| 09/04/2015 | DB3 | 4.4185 | 0.2629 | 775 |
| 09/04/2015 | DB4 | 3.3944 | 0.1579 | 1162 |
| 09/04/2015 | DB5 | 4.4653 | 0.1949 | 1003 |
| 09/04/2015 | DB8 | 2.4667 | 0.1695 | 2158 |
| Mean ± SD | DB group | 3.663 ± 0.739 | 0.218 ± 0.053 | 1361 ± 804 |
| 17/04/2015 | DA9 | 4.7199 | 0.4209 | 7,072 |
| 17/04/2015 | DA10 | 4.9527 | 0.64 | 9,631 |
| 17/04/2015 | DA12 | 5.1876 | 0.4925 | 4,987 |
| 17/04/2015 | DA13 | 5.0759 | 0.4646 | 6,254 |
| 17/04/2015 | DA15 | 4.987 | 0.547 | 5,311 |
| 17/04/2015 | DA16 | 5.6915 | 0.4907 | 6,823 |
| Mean ± SD | DA group | 5.102 ± 0.328 | 0.509 ± 0.076 | 6680 ± 1611 |
| 12/05/2015 | C17 | 2.5477 | 0.4564 | BLOQ |
| 12/05/2015 | C22 | 1.3976 | 0.0621 | BLOQ |
| 12/05/2015 | C26 | 2.6005 | 0.1144 | BLOQ |
| 12/05/2015 | C27 | 2.7487 | 0.0934 | BLOQ |
| 12/05/2015 | C29 | 2.4145 | 0.0905 | BLOQ |
| 12/05/2015 | C31 | 2.1761 | 0.1192 | BLOQ |
| 12/05/2015 | C31 | 2.1761 | 0.1192 | BLOQ |
| Mean ± SD | C group | 2.314 ± 0.489 | 0.156 ± 0.149 | BLOQ |

SD: standard deviation; BLOQ: below the limit of quantification.

**Table S2.** List of level 2 enriched gene ontology (GO) terms for differentially expressed genes in biological process (BP), molecular function (MF) and cellular component (CC) categories.

| **GO ID** | **GO Name** | **GO Type** | **UP/DOWN** | **FDR** | **Fold Enrichment** |
| --- | --- | --- | --- | --- | --- |
| GO:0008152 | metabolic process | BP | UP | 6.54 × 10^−62^ | 1.56 |
| GO:0009987 | cellular process | BP | UP | 3.18 × 10^−42^ | 1.47 |
| GO:0065007 | biological regulation | BP | DOWN | 2.74 × 10^−10^ | 1.37 |
| GO:0050789 | regulation of biological process | BP | DOWN | 8.16 × 10^−9^ | 1.37 |
| GO:0023052 | signaling | BP | DOWN | 8.71 × 10^−5^ | 1.37 |
| GO:0002376 | immune system process | BP | DOWN | 9.25 × 10^−5^ | 2.36 |
| GO:0050896 | response to stimulus | BP | DOWN | 4.97 × 10^−4^ | 1.27 |
| GO:0071840 | cellular component organization or biogenesis | BP | UP | 8.02 × 10^−4^ | 1.33 |
| GO:0015976 | carbon utilization | BP | UP | 1.01 × 10^−3^ | 3.88 |
| GO:0051179 | localization | BP | UP | 1.82 × 10^−2^ | 1.19 |
| GO:0003824 | catalytic activity | MF | UP | 7.67 × 10^−75^ | 1.63 |
| GO:0005198 | structural molecule activity | MF | UP | 8.31 × 10^−8^ | 1.96 |
| GO:0005488 | binding | MF | DOWN | 1.37 × 10^−7^ | 1.15 |
| GO:0060089 | molecular transducer activity | MF | DOWN | 2.54 × 10^−6^ | 2.22 |
| GO:0140110 | transcription regulator activity | MF | DOWN | 2.64 × 10^−6^ | 1.85 |
| GO:0038024 | cargo receptor activity | MF | DOWN | 3.85 × 10^−6^ | 2.98 |
| GO:0098772 | molecular function regulator | MF | UP | 9.92 × 10^−5^ | 1.70 |
| GO:0005215 | transporter activity | MF | UP | 3.64 × 10^−4^ | 1.46 |
| GO:0044464 | cell part | CC | UP | 4.77 × 10^−28^ | 1.51 |
| GO:0005623 | cell | CC | UP | 7.70 × 10^−27^ | 1.49 |
| GO:0032991 | protein‐containing complex | CC | UP | 5.84 × 10^−25^ | 1.66 |
| GO:0016020 | membrane | CC | UP | 7.93 × 10^−16^ | 1.41 |
| GO:0043226 | organelle | CC | UP | 5.89 × 10^−14^ | 1.44 |
| GO:0044425 | membrane part | CC | UP | 3.58 × 10^−8^ | 1.35 |
| GO:0044422 | organelle part | CC | UP | 2.12 × 10^−6^ | 1.40 |
| GO:0044421 | extracellular region part | CC | DOWN | 2.22 × 10^−2^ | 1.87 |

UP/DOWN indicates if most of the genes in the category were up‐ or down‐regulated.
